# Optimization of transmission-scan time for the FixER method: a MR-based PET attenuation correction with a weak fixed-position external radiation source

**DOI:** 10.1186/2197-7364-1-S1-A31

**Published:** 2014-07-29

**Authors:** Hiroshi Kawaguchi, Yoshiyuki Hirano, Jeff Kershaw, Eiji Yoshida, Takahiro Shiraishi, Mikio Suga, Takayuki Obata, Hiroshi Ito, Taiga Yamaya

**Affiliations:** Molecular Imaging Center, National Institute of Radiological Sciences, Chiba, Japan; Research Center for Charged Particle Therapy, National Institute of Radiological Sciences, Chiba, Japan; Center for Frontier Medical Engineering, Chiba University, Kragujevac, Japan

In recent work, we proposed an MRI-based attenuation-coefficient (μ-value) estimation method that uses a weak fixed-position external radiation source to construct an attenuation map for PET/MRI. In this presentation we refer to this method as FixER, and perform a series of simulations to investigate the duration of the transmission scan required to accurately estimate μ-values.

In FixER, a segmented anatomical image and transmission data acquired with a fixed-position radiation source are needed to obtain µ-values for each segment. FixER has the advantage that segmentation reduces the number of unknowns and tissue identification is unnecessary. First, the geometry of a whole body PET scanner was replicated in virtual space, and a T1-weighted head image was segmented into air, bone, brain, and soft tissues other than brain. Simulated PET transmission scans were performed with the fixed-position ^22^Na point source (1MBq) to obtain the intensities with (**I**) and without (**I**_0_) a digital phantom made from the segmented tissues. The blank scan to obtain **I**_0_ was performed for 180 secs. The path length of each tissue was then calculated from the segmented image and straight lines drawn from the source to each crystal. Finally, the μ-values of each tissue type were calculated based on Lambert-Beer's law.

All µ-values converged with increasing scan time. The µ-values for brain, bone and soft tissues were 0.101, 0.120 and 0.095 cm^-1^, respectively, for a 180 secs transmission scan. Using these values as points of reference, it was seen that a transmission scan of at least 46 secs is needed to estimate all µ-values within an accuracy of 3%.

The optimal transmission-scan time for FixER was investigated for head imaging. In future work it is planned to also optimize the position and number of point sources for various body regions and numbers of segments.

